# Recovery of Benthic Megafauna from Anthropogenic Disturbance at a Hydrocarbon Drilling Well (380 m Depth in the Norwegian Sea)

**DOI:** 10.1371/journal.pone.0044114

**Published:** 2012-10-08

**Authors:** Andrew R. Gates, Daniel O. B. Jones

**Affiliations:** Ocean Biogeochemistry and Ecosystems Group, National Oceanography Centre, Southampton, United Kingdom; Université du Québec à Rimouski, Canada

## Abstract

Recovery from disturbance in deep water is poorly understood, but as anthropogenic impacts increase in deeper water it is important to quantify the process. Exploratory hydrocarbon drilling causes physical disturbance, smothering the seabed near the well. Video transects obtained by remotely operated vehicles were used to assess the change in invertebrate megafaunal density and diversity caused by drilling a well at 380 m depth in the Norwegian Sea in 2006. Transects were carried out one day before drilling commenced and 27 days, 76 days, and three years later. A background survey, further from the well, was also carried out in 2009. Porifera (45% of observations) and Cnidaria (40%) dominated the megafauna. Porifera accounted for 94% of hard-substratum organisms and cnidarians (Pennatulacea) dominated on the soft sediment (78%). Twenty seven and 76 days after drilling commenced, drill cuttings were visible, extending over 100 m from the well. In this area there were low invertebrate megafaunal densities (0.08 and 0.10 individuals m^−2^) in comparison to pre-drill conditions (0.21 individuals m^−2^). Three years later the visible extent of the cuttings had reduced, reaching 60 m from the well. Within this area the megafaunal density (0.05 individuals m^−2^) was lower than pre-drill and reference transects (0.23 individuals m^−2^). There was a significant increase in total megafaunal invertebrate densities with both distance from drilling and time since drilling although no significant interaction. Beyond the visible disturbance there were similar megafaunal densities (0.14 individuals m^−2^) to pre-drilling and background surveys. Species richness, Shannon-Weiner diversity and multivariate techniques showed similar patterns to density. At this site the effects of exploratory drilling on megafaunal invertebrate density and diversity seem confined to the extent of the visible cuttings pile. However, elevated Barium concentration and reduced sediment grain size suggest persistence of disturbance for three years, with unclear consequences for other components of the benthic fauna.

## Introduction

Exploratory hydrocarbon drilling activities are increasing in deeper water [1], [2] and in more environmentally sensitive areas [3]. Environmental impacts associated with offshore exploration drilling include the discharge of cuttings on to the seabed [4], discharge of produced water [5] and the possibility of a major blow out or oil spill [6]. By their nature blow outs and oil spills are unpredictable events, but disturbance from cuttings is well regulated and monitored, providing a useful opportunity to study disturbance in inaccessible and normally quiescent deep waters.

In modern best-practice exploration drilling, disturbance to the seabed at well locations results from the discharge of a mixture of drill cuttings and water-based drilling mud (fluid used to lubricate the drill bit and maintain the structural integrity of the well).This occurs during the initial phase of drilling when the widest diameter sections of the hole are drilled (the “top-hole”), before the marine riser and blow-out preventer (BOP), a large metal structure sitting on top of the well, are deployed. This disturbance is characterized by a combination of physical smothering of the seabed, associated changes in sediment structure, and the potential toxic effects of exposure to the chemical constituents of the mud used in the drilling process [7], [8], [9]. Barite is often added as a weighting agent in drilling mud so barium is a frequently used tracer for drilling disturbance [10], [11]. After deployment of the BOP the cuttings and mud are re-circulated to the surface, cleaned and discarded from the rig. In contrast to this practice, older methods of exploration drilling discharged greater quantities of oil-based drilling mud and cuttings to the seabed.

Exploration drilling disturbance initially results in reduced abundance and diversity of the meiofaunal [12], macrofaunal [13], [14] and megafaunal [4], [15] components of benthic communities. The deposition of cuttings will also affect sediment bacteria, which can comprise up to 90% of benthic biomass [16]. Reduced benthic diversity, in turn, may result in reduced ecosystem functioning [17]. In addition, there is some experimental evidence that drilling disturbance changes overall ecosystem functioning. Biogeochemical fluxes from the sediment (leading to oxygen depletion in the sediment) were altered immediately after addition of cuttings, and bioturbation inhibited by increased sedimentation [8], [18], [19].

In the north-east Atlantic, where water-based drilling mud is used, exploration drilling usually has an impact on the seabed, visible in remote video survey, extending 100 to 200 m from the well. This results in reduced sediment heterogeneity and significant reductions in megafaunal abundance and diversity shortly after the disturbance [4], [20]. According to older studies, which report on disturbance from oil-based drilling mud, hydrocarbon drilling in shallower water leads to altered sediment characteristics with resultant changes to macrobenthic communities over larger areas [11], [20], [21]. Even in more accessible shallower areas it is unclear how long the effects of such disturbance persist [22] and few studies of recovery from any form of anthropogenic disturbance have been carried out in deep water [23], [24].

Recovery typically implies the return of an ecosystem to pre-disturbance conditions as a result of the operation of homeostatic ecological control mechanisms [25]. Recovery is a complex phenomenon involving various spatially and temporally dynamic biotic and abiotic changes. The recovered ecosystem may be altered in some way from its original state, for example in terms of function, structure, species composition or diversity [25].

The benthic megafauna includes those organisms over 1 cm that inhabit the sediment-water interface [26]. Benthic megafaunal organisms play a key role in the functioning of deep-sea ecosystems [27]. Through their actions such as burrowing and feeding they redistribute sediment and influence local scale biogeochemistry [28], [29]. The presence of sessile forms may influence habitat heterogeneity [30]. The megafauna may be affected in several ways by drilling disturbance. For example, physical smothering has been shown to induce increased stress protein expression in motile forms [31] while sessile suspension feeding organisms may also be negatively affected by sedimentation [32].

The well-documented and relatively accessible nature of exploration drilling disturbance provides a valuable opportunity to investigate the process of recovery of benthic megafauna in deeper water. Through the SERPENT project [33] a time-series study of the benthic invertebrate megafauna was carried out around an exploration well at the Morvin field in the Norwegian Sea. Surveys were conducted before drilling, and 27 days, 76 days and three years after drilling and addressed four objectives: 1) to describe the megafaunal species diversity and abundance at the Morvin location, 2) to identify the temporal change in the visible extent of drill cuttings disturbance, 3) to carry out a local-scale, time-series assessment of recovery of benthic megafaunal invertebrates from hydrocarbon drilling disturbance, 4) to use evidence of bioturbation as an indicator of ecosystem function along a disturbance gradient. These objectives are designed to test the hypothesis that over a period of three years physical and biological processes redistribute drill cuttings and water based mud to an extent that megafaunal organism abundance and diversity can recover after an initial physical disturbance from exploration drilling in deeper water.

## Methods

### Ethics statement

No specific permits were required for the described field studies. The site was part of Statoil's production licence 134b and subject to oil drilling activities. No invertebrate megafauna specimens were collected as the work was carried out using video techniques.

### Study location

**Figure 1:**
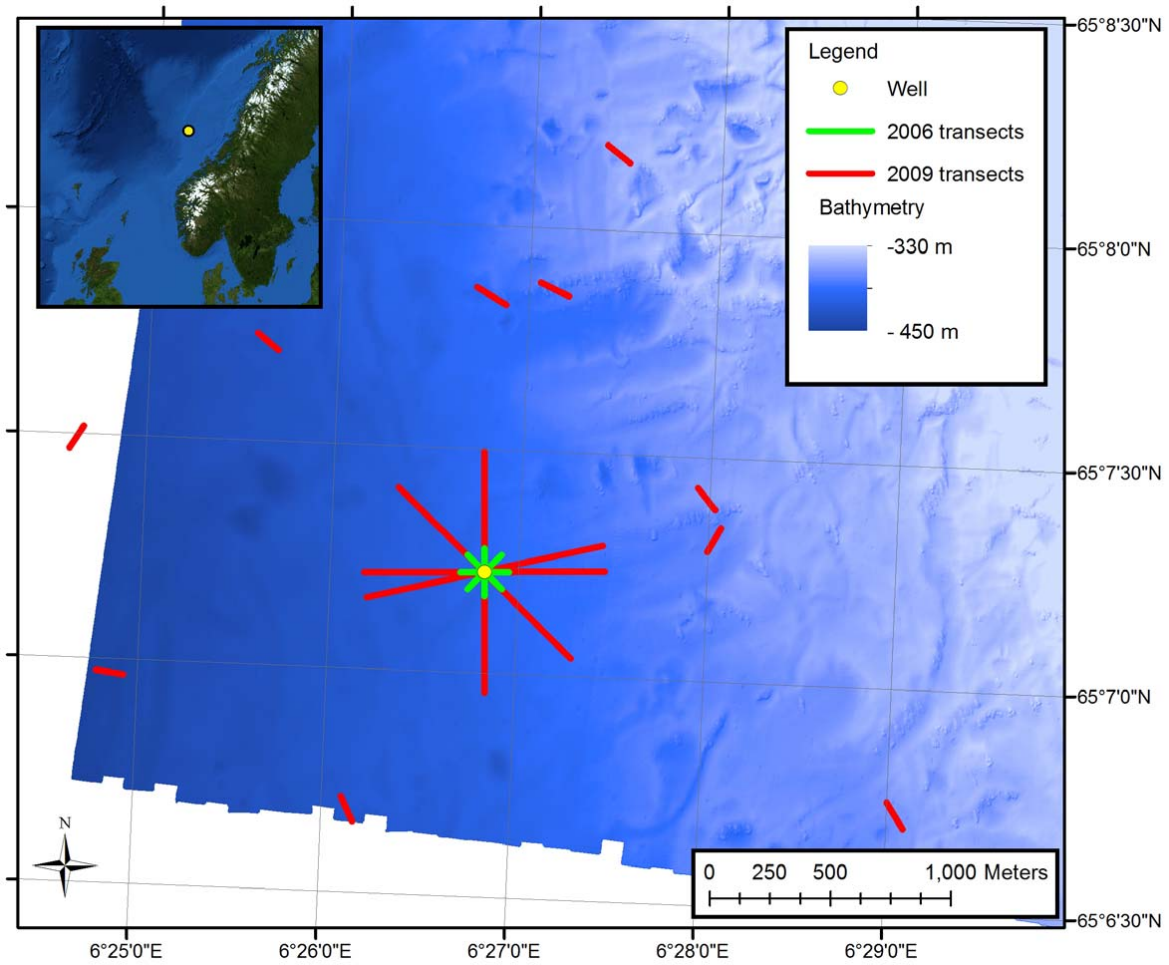
The Morvin survey design. The 2009 video transect survey is shown in red. Previous surveys were at the same location with 100 m video transects radiating from the well and are shown in green. The location of the Morvin field in the Norwegian Sea is shown as an inset.

The Morvin field is located on the continental slope of the Norwegian Sea (Figure 1). On 24^th^ March 2006 drilling commenced on an exploration hydrocarbon well from the semi-submersible drilling rig *West Alpha* in 380 m water depth; position 380172 E, 7224481 N. Positional information was recorded in Universal Transverse Mercator (UTM) zone 32 N based on the European Datum 1950 (ED50).

### Data collection

#### Video surveys

Three video transect surveys were carried out in 2006 using an Oceaneering Hydra Magnum 041 work-class drill-support Remotely Operated Vehicle (ROV) launched directly from the West Alpha. Each survey comprised eight transects, approximately 100 m in length, limited by ROV tether length (owing to launch from the stationary drilling rig). Surveys were carried out one day before (23^rd^ March 2006), 27 days after (21^st^ April 2006) and 76 days (9^th^ July 2006) after drilling. The straight-line transects radiated from the well location in 8 directions (0, 45, 90, 135, 180, 225, 270, 315°: Figure 1). Transects conducted before drilling followed a set heading (using the ROV gyrocompass) from a buoy marking the intended well position. Distance from the well was estimated from the amount of ROV tether unwound. After drilling ROV sonar was used to improve navigational precision and transects were flown towards the BOP (a clear sonar target). The ROV was flown at a speed of approximately 0.2 m s^−1^ with the standard-definition colour video camera (Kongsberg OE1366) approximately 1 m above the seabed. The camera was positioned at an angle of 18° from horizontal (the maximum angle possible without viewing the ROV frame) with the zoom set to maximum wide angle. Transect width (mean of 1.0 m; max variation±0.2 m) was calculated from the camera acceptance angles and verified following Jones et al. (2006). A digital stills camera (Kongsberg OE14-108) was used to obtain high-resolution photographs of organisms for species identification in separate, opportunistic surveys. The pre-drilling SW transect was omitted from further analysis owing to poor visibility.

Over the 3^rd^ to 4^th^ May 2009, more than three years after drilling commenced, an additional video survey was carried out from the vessel *Acercy Petrel* equipped with the Acergy Solo MKII survey class ROV. Four video transects of 1 km length were carried out, crossing the well at their mid point. For comparison, ten reference transects were also taken (Figure 1). These were 100 m in length, between 1 and 3 km from the well. Starting points and headings for the reference sites were randomly selected. The Morvin area had been the subject of extensive deep-water coral reef mapping and studies of seabed fluid flow [34], [35]; thus any reference transects located near possible reef features were rejected and another random starting point and heading generated.

Recording of the transects began and ended 20 m beyond the planned positions to ensure that the correct altitude and speed were attained before the intended start/finish point. The ROV was flown at approximately 0.3 m s^−1^ with the camera height of approximately 2.5 m above the seabed. The colour video camera (IMENCO Z 1051) was as close to vertical as possible at an angle of 24° below the horizontal with the zoom set to maximum wide angle (mean transect width of 2.6 m; max variation±0.3 m). UTM positional data (from Ultra-Short Baseline Navigation) were continually recorded. The greater ROV altitude in this survey is because of differences in equipment associated with the survey carried out from a ship in contrast to the earlier surveys which were carried out from a drilling rig and may cause some variation in both species density and diversity measurements.

The four surveys described above will be referred to as “Pre” (1 day before drilling), “Post 1” (27 days after drilling commenced), “Post 2” (76 days after drilling) and “Post 3” (three years after drilling). Reference sites studied three years after drilling are referred to as “R” sites.

#### Additional data collection

Sediment samples were collected using ROV push corers before and after drilling. Before drilling, single samples were collected from the well location at approximately 50 and 100 m north of the well. After drilling (21^st^ April 2006) single samples were collected at 10 m and 100 m north east and west of the well. The samples were retrieved to the surface, the depth of an visible drill cuttings measured and the top 50 mm retained and frozen.

Five graduated marker buoys were deployed around the well before drilling commenced. The marker buoys were placed at eight metres north, east and west of the well and at 50 m and 100 m north east of the well. Observations of sediment accumulation around the buoys were made using the ROV at intervals during the drilling programme. The buoys were removed from the seabed at the end of the drilling programme in 2006.

In 2009 three replicate sediment samples were collected using ROV push corers at 25 and 50 m from the well on four headings (N, NW & SW). On all headings the samples were divided into 0–20, 20–40 and 40–60 mm sections and preserved by freezing. It was not possible to collect the planned samples at 100 m from the well because of time limitations.

### Video data analysis

In all cases, video was replayed at half speed and every individual animal was counted and its position recorded as it passed the bottom of the screen. Colonial organisms were counted as single individuals. Megafaunal organisms were identified to the lowest possible taxonomic level. Where species identification was not possible, operational taxonomic units (OTU) were used. Fish were excluded from analysis of benthic abundance data because of their motility and tendency of some species to follow the ROV. Megafaunal density was calculated from abundances divided by the area of the transect section (transect section length multiplied by image width). Features on the seabed such as rocks and burrows were recorded and all data were plotted in a geographic information system using the software ArcGIS (version 9.3).

The distribution of drill cuttings was assessed visually from the video footage. Disturbed sediment was recognized on the basis of its characteristically pale colour and absence of visible evidence of bioturbation (Table S1). The boundaries of the disturbed area were identified and mapped. Megafaunal datasets were extracted from these zones in ArcGIS for comparison of the disturbed zones with other areas.

Data for each well-site transect were split into 100 m distance zones. In the post-drilling surveys part of the 0–100 m zone was visibly disturbed, so this sample unit was split into two sections “Disturbed” and “Beyond Disturbance” in order to identify the effects of disturbance at the highest resolution possible with video observations. For statistical analysis the pre-disturbance transects were split into the same sections as described above (based on the disturbance extent in Post 1 in 2006) so that the densities of fauna in the pre-drilling samples were properly compared in the statistical model. Results were presented based on the disturbance zones rather than consistent distance zones in order to identify the impact after three years.

To describe abundance, both total density and density of organisms associated with different substrata were calculated. A range of diversity indices were calculated to assess both the evenness and species richness elements of diversity [36]. Sampling units were of variable area so species richness (*S*) was rarefied to 50 individuals (*ES_(50)_*). Evenness was calculated as Pielou Evenness (*J′*). In addition, the widely-used Shannon-Wiener Index (*H′*) was presented to allow comparison with other studies. These measures were calculated using the software package PRIMER v.6 [37].

Three generalized linear (GLM) statistical models were independently developed [38] to examine whether the density (no. m^−2^) of total, sessile and motile megafauna at Morvin could be explained using the explanatory variables distance and year. Random sites were coded with a distance of >1000 m from drilling and included in all analysis. All explanatory variables were treated as categorical data. The model was fitted with quasi-Poisson errors using the R function GLM and the ANOVA function of the R package CAR (companion to applied regression) [39] in the R programming environment [40].

The megafaunal assemblage composition was investigated using multivariate analyses. A fourth root transformation was applied to buffer the influence of dominant taxa and similarities were calculated using Bray-Curtis coefficients [41]. The similarity values were subjected to both classification (hierarchical group-average clustering) and ordination (non-metric multi-dimensional scaling, MDS) using the PRIMER software. The difference in the megafaunal assemblage composition was assessed using two-way permutational multivariate analysis of variance (PERMANOVA) [42] with distance zones and survey time as factors. PERMANOVA was implemented using the R package Vegan [43].

In addition to the megafauna, structures on the seabed were documented. Rocks were counted and used in later analysis to document the background environment. Conspicuous burrows in the sediment (likely decapods, *Geryon* sp. – Figure S1) were also counted as an indicator of bioturbation activity along the disturbance gradient.

### Recovery

Response Y, which represents recovery of the benthic environment after disturbance [44], was calculated based on the percentage change from mean “pre-drill” values of the following indices of diversity: mean motile organism density, sessile organism density, species richness, evenness, Shannon Wiener diversity and Bray-Curtis similarity. Response Y is the percentage difference between impacted and control sites. In order to prevent a right-skewed distribution [44], it is presented transformed as follows (where X is the percentage difference from the pre-drill survey):
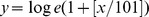
Variation in response Y was tested using two-way ANOVA on ranks with the factors distance and year using the R package.

### Environmental data

Chemical (heavy metals) and particle size distribution analyses were conducted on the sediment samples. Heavy metals analysis (Cd, Pb, As, Se, Sn) was carried out using atomic absorption spectroscopy (Perkin Elmer SIMAA 6000). The method applied was in accordance with Norwegian standard NS4770 and consisted of a partial acidic extraction using 7 NHNO3 in an autoclave. Mercury was analysed according to the same standard but using a different instrument (CETAC M-6000A Hg Analyzer). Thirty other elements were analysed according to the same standard using ICP-AES (Perkin Elmer Optima 4300 Dual View). Particle size distributions were determined using a Coulter LS200 instrument in the range 0.4–2000 µm.

During the surveys depth and temperature were measured using a ROV-mounted sensor (Paroscientific Digiquartz® 8 series).

## Results

### The background environment

The well was located at 380 m depth. There was no appreciable depth variation within 100 m of the well but beyond this, in the 2009 survey area (well site and reference video transects) depth varied between 362 m and 397 m. The predominantly flat sediment was punctuated by small rocks providing some hard substratum. Decapod burrows in the soft sediment were an important feature of the environment. Seabed water temperature was 7.4°C on both 20^th^ April 2006 and 3^rd^ May 2009. Salinity was 35.5 on the same date in 2009, but was not measured in 2006.

**Figure 2:**
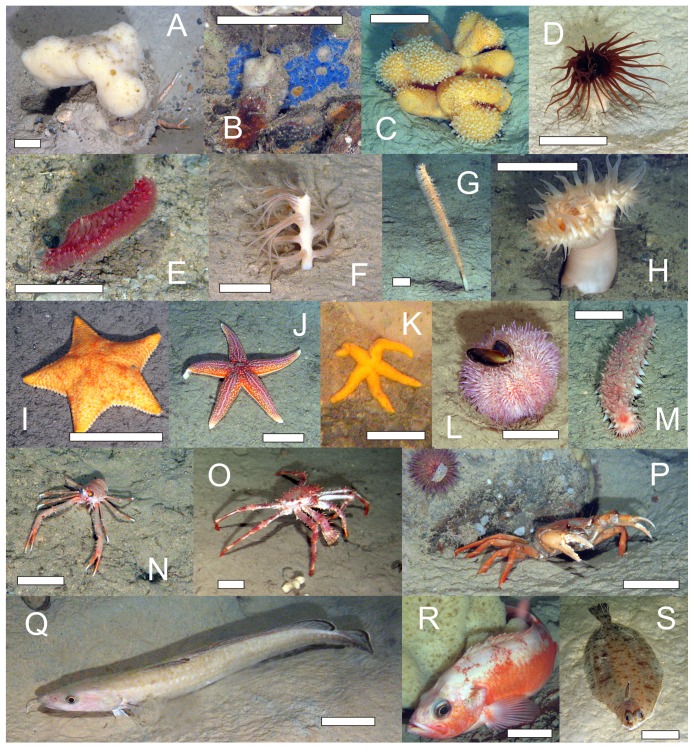
Examples of the megafaunal taxa observed at Morvin. A: *Mycale* sp., B: *Hymedesmia* sp., C: *Alcyonium* sp., D: *Cerianthus* sp., E: *Pennatula phosphorea*, F: *Kophobelemnon stelliferum*, G: *Funiculina* sp., H: *Bolocera* sp., I: *Porania* sp., J: *Asterias rubens*, K: *Henricia* sp., L: *Echinus* sp., M: *Parastichopus tremulus*, N: *Munida* sp., O: *Lithodes* sp., P: *Geryon* sp., Q: *Molva molva*, R: Sebastidae, S: *Glyptocephalus cynologus*. Scale bar on images represents 50 mm.

The invertebrate megafauna observed during the background quantitative video surveys (Pre-drill and R transects) comprised 27 distinct taxa with a total density of 0.22 m^−2^ (examples shown in Figure 2 and listed in Table 1). Additional taxa were observed across all the disturbance transects. The megafauna was dominated by Porifera (44.5% of total fauna) and Cnidaria (40.6%). The Echinodermata (11.6%) were also important. Of the Cnidaria, soft-sediment dwelling pennatulid octocorals were most abundant and were represented by four distinct taxa, of which *Kophobelemnon stelliferum* was the most common (24.5% of all observations). There were nine distinct poriferan taxa, which were predominantly attached to hard substrates; *Phakellia* sp. (13.5%) and the unidentified “tiny white sponge” (9.2%) were the most abundant. The echinoderms were dominated by the deposit-feeding holothurian *Parastichopus tremulus* (8.3%).

**Table 1:** Mean megafaunal taxon density (per 100 m^−2^) from video observations before, during and after the drilling operations at increasing distance from the well.

		Background		Post drill 1		Post drill 2		Post drill 3					
likely species/morphotype	Substratum	Pre	R	Dist	Beyond	Dist.	Beyond	Dist.	Beyond	100–200 m	200–300 m	300–400 m	400–500 m
*Phakellia* sp. 1 †	H	3.18	2.97	0.37	0.00	3.67	1.99	0.16	1.09	1.30	1.32	0.61	0.62
small spherical white sponges	H	2.32	2.04	0.24	0.16	0.09	1.50	0.52	1.20	1.97	1.57	1.48	1.55
*Mycale* sp.†	H	0.42	2.51	0.12	0.16	0.09	0.99	0.35	0.59	1.01	1.49	0.86	0.84
*Haliclona* sp.	H	1.23	0.94	0.12	0.37	0.81	0.49	0.16	0.69	0.71	1.01	0.69	0.92
Encrusting white sponge	H	1.60	0.65	0.24	0.00	0.09	0.27	0.47	0.48	0.45	0.51	0.10	0.20
*Stylocordyla borealis*	S	0.49	0.51	0.00	0.00	0.00	0.12	0.00	0.41	1.01	0.79	0.55	0.68
*Hymedesmia* sp.†	H	0.42	0.04	0.00	0.00	0.00	0.27	0.00	0.20	0.20	0.20	0.00	0.05
*Axinella* sp.†	H	0.26	0.36	0.00	0.00	0.09	0.27	0.00	0.00	0.00	0.00	0.00	0.00
*Phakellia sp.* 2	H	0.00	0.08	0.00	0.00	0.00	0.31	0.00	0.00	0.00	0.00	0.00	0.00
*Kophobelemnon stelliferum* †	S	5.12	5.63	4.01	6.91	1.93	8.52	0.87	3.99	3.85	3.65	2.92	2.90
small straight pennatulid	S	0.83	1.39	0.18	0.48	1.39	1.80	0.63	1.08	1.25	1.30	1.57	0.90
*Funiculina* sp.†	S	0.14	1.74	0.00	0.37	0.09	0.63	0.00	0.42	0.51	0.36	0.28	0.78
*Cerianthus* sp.†	S	0.00	0.39	0.35	0.21	0.19	0.43	0.00	0.53	0.14	0.10	0.29	0.50
*Bolocera* sp.†	H	0.53	0.31	0.21	0.48	0.20	0.47	0.00	0.24	0.00	0.20	0.15	0.14
*Pennatula phosphorea* †	S	0.39	0.36	0.12	0.43	0.09	0.52	0.00	0.08	0.15	0.16	0.14	0.00
*Alcyonium* sp.†	H	0.00	0.36	0.00	0.00	0.09	0.27	0.19	0.08	0.05	0.10	0.05	0.00
red cnidarian	H	0.00	0.00	0.00	0.00	0.00	0.00	0.00	0.00	0.16	0.05	0.05	0.00
*Lophelia pertusa*	H	0.00	0.04	0.00	0.00	0.00	0.00	0.00	0.00	0.00	0.00	0.05	0.10
*Colus sp.*	G	0.14	0.08	0.00	0.00	0.00	0.36	0.00	0.00	0.05	0.00	0.00	0.00
*Geryon* sp.†	S	0.28	0.08	1.31	2.14	0.00	1.30	0.00	0.25	0.05	0.05	0.04	0.00
*Pandalus* sp.†	G	0.42	0.11	0.00	0.00	0.37	0.88	0.13	0.33	0.35	0.15	0.05	0.14
*Lithodes* sp.†	G	0.00	0.00	0.00	0.00	1.18	0.35	0.00	0.00	0.00	0.05	0.00	0.00
*Munida* sp.†	G	0.14	0.04	0.00	0.00	0.00	0.18	0.74	0.12	0.05	0.11	0.05	0.05
Bryozoan	H	0.00	0.00	0.00	0.00	0.00	0.12	0.00	0.16	0.21	0.11	0.10	0.19
*Nipponemertes* sp.	S	0.00	0.00	0.00	0.00	0.00	0.00	0.00	0.10	0.00	0.05	0.05	0.00
echiuran*	S	0.00	0.00	0.00	0.00	0.00	0.00	0.00	0.00	0.00	0.00	0.00	0.00
*Parastichopus tremulus* †	S	2.62	1.68	0.00	0.46	0.00	0.52	0.69	1.18	1.80	1.25	1.39	1.82
*Henricia* sp.†	G	0.00	0.20	0.34	0.00	0.09	0.53	0.27	0.08	0.14	0.15	0.10	0.05
*Ceramaster* sp.	G	0.26	0.23	0.00	0.00	0.00	0.28	0.00	0.11	0.14	0.21	0.30	0.36
*Asterias rubens* †	G	0.00	0.00	0.00	0.00	0.00	0.76	0.00	0.00	0.00	0.00	0.00	0.00
*Porania* sp.†	G	0.00	0.20	0.00	0.00	0.00	0.16	0.00	0.00	0.14	0.00	0.05	0.05
*Hippasteria* sp.	G	0.00	0.00	0.00	0.00	0.00	0.00	0.00	0.00	0.00	0.10	0.00	0.00
*Crossaster* sp.*	G	0.00	0.00	0.00	0.00	0.00	0.00	0.00	0.00	0.00	0.00	0.00	0.00
*Cidaris cidaris*	G	0.14	0.12	0.00	0.00	0.00	0.00	0.00	0.00	0.00	0.00	0.05	0.00
*Echinus* sp.†	G	0.14	0.08	0.00	0.00	0.09	0.37	0.00	0.12	0.10	0.15	0.26	0.21
Indet 2	H	0.00	0.00	0.00	0.00	0.00	0.00	0.00	0.00	0.05	0.00	0.00	0.00
Total density		21.05	23.11	7.63	12.18	10.57	24.68	5.19	13.52	15.86	15.17	12.25	13.05

* = species that were observed but not recorded, either outside of survey area or large motile organisms intentionally excluded to prevent over estimation of abundance.

† = higher resolution still photograph collected, otherwise only recorded from video footage. H = hard-substratum organisms, S = soft-substratum organisms, G = generalists, organisms seen on both hard and soft substrata.

### Species diversity and community composition at background sites

**Figure 3:**
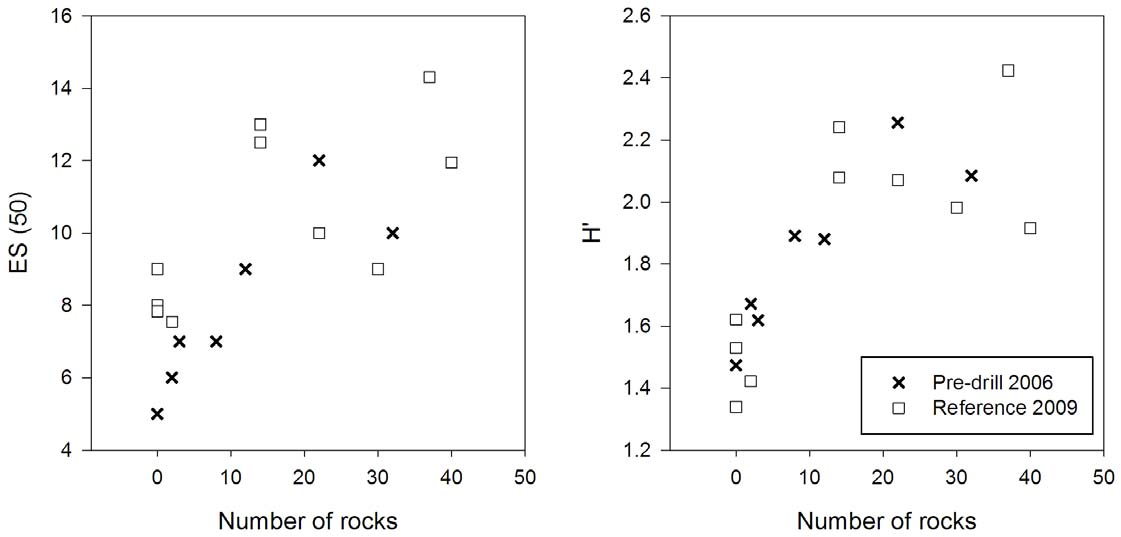
Habitat heterogeneity and species diversity at background sites. The relationship between the number of rocks observed in video transects and two indices of megafaunal invertebrate species diversity for the 2006 Pre-drill video survey and 2009 Reference sites (randomly selected undisturbed locations) (left; rarefied species richness *ES_(50)_*, right; Shannon-Weiner Index *H′*).

Univariate analysis showed no significant difference in diversity (density, *S*, *H′*, *J*) between the R sites and Pre sites (assessing temporal variation between 2006 and 2009). There was also no significant difference in multivariate community composition among the background (Pre and R) transects (PERMANOVA, F_(1)_ = 1.405, p = 0.171). However, assessing fine-scale spatial heterogeneity, there was a positive relationship between the number of rocks in the background transects and species richness and diversity (linear regression; Rarefied species richness: R^2^ = 0.51, ANOVA, F_(1, 15)_ = 15.58, p<0.001, Shannon Wiener species diversity: R^2^ = 0.60, ANOVA, F_(1, 15)_ = 22.47, p<0.001; Figure 3). Rocks were unevenly distributed throughout the survey area and their presence increased the between-transect variation in measures of total density and diversity.

### Physical disturbance

**Figure 4:**
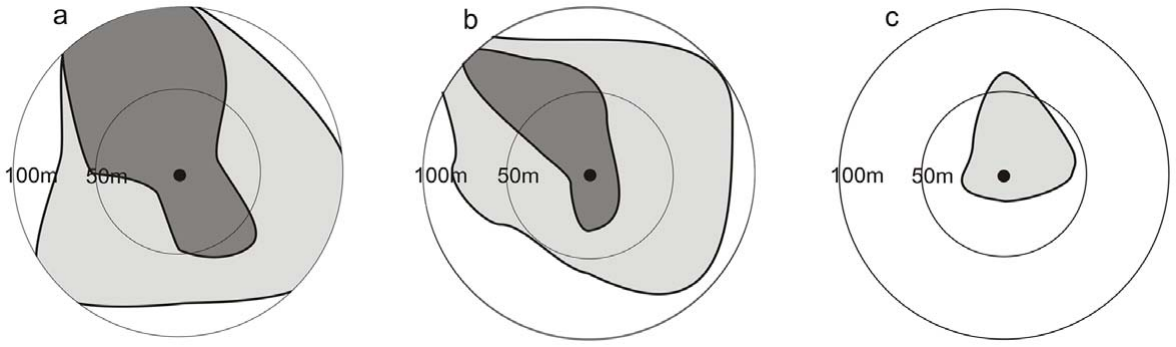
Physical disturbance at Morvin. Representation of the visible horizontal extent of drilling disturbance at Morvin: a) Post 1, b) Post 2, c) Post 3. The filled black circles in the centre represent well position, dark grey = complete coverage of sea bed with drill cuttings; light grey = partial coverage.

The well was drilled in April 2006, resulting in the discharge of 192000 kg of barite drilling mud to the seabed. Discharge to the seabed was only from the top-hole (42″ and 36″ diameter sections). In addition, 77000 kg of barite were discharged to the sea surface from the 17.5″ section (Aas 2008, unpublished report). This resulted in a disturbance to the seabed with visible cuttings extending beyond 100 m in some directions in the two post-drilling surveys in 2006 (Figure 4). The visible extent of this disturbance decreased from >26600 m^2^ in 2006 to 3500 m^2^ by 2009. Seventy-six days after drilling, the cuttings reached 400 mm in thickness close to the well. At 50 m distance the thickness of the deposit was considerably less (<50 mm) but still evident as a layer at the surface of the push cores (Table 2). In Post drill 2 the mean sediment barium (Ba) concentration (5450 mg kg^−1^) was elevated above the pre-drilling concentration (150 mg kg^−1^) and Norwegian Continental Shelf background levels (4.6–554 mg kg^−1^) (SINTEF unpublished report). Three years later (Post 3), the mean sediment surface Ba concentration remained high at 25 and 50 m from the well (6133 and 6291 mg kg^−1^ in the top 20 mm) but decreased with depth in the sediment (3283 and 547 mg kg^−1^ at 40–60 mm). There were significant differences in the Ba concentration of the sections taken from different depths in the sediment at both 25 m (ANOVA, F_(2, 21)_ = 4.02, p<0.05) and 50 m (Kruskal-Wallis H_(2)_ = 15.97, p<0.001). Sediment particle size was affected by the deposition of drilling mud and cuttings. The percentage of particles under 69 µm (% fines) increased in the near-well samples taken in Post 2 in comparison to the Pre samples and those taken further from the disturbance in Post 2. In Post 3, the % fines remained high in the surface sediment at both 25 and 50 m from the well. There was a reduction in % fines with depth in the sediment, reaching values similar to Pre-drill at 20–40 and 40–60 mm depth at 50 m from the well.

**Table 2:** Measurements of the depth of drill cuttings from graduated marker buoys and Barium concentration and sediment particle size from push core samples taken at the Morvin site during the Pre, Post 2 and Post 3 surveys.

Survey	Section (mm)	Distance (m) from well	Depth of cuttings (mm)	Ba (mg kg^−1^)	Sediment particle size; % fines (<69 µm)
Pre	0–50	0	0	150	53.6
Pre	0–50	50	0	n/a	37.9
Pre	0–50	100	0	n/a	38.4
Post 2	0–50	0–10	400	5450 (±1202)	80.2 (±3.4)
Post 2	0–50	100	<50	230	45.4 (±4.3)
Post 3	0–20	25		6133(±1332)	76.9 (±15.6)
Post 3	20–40	25		4791(±1998)	63.8 (±26.9)
Post 3	40–60	25		3283(±2525)	60.3 (±27.9)
Post 3	0–20	50		6291(±1505)	58.1 (±19.7)
Post 3	20–40	50		1991(±2438)	41.3 (±9.6)
Post 3	40–60	50		547(±454)	37.1 (±6.0)

Figures in parentheses are standard deviation. For Post 2 samples n = 3 and for Post 3 samples n = 8.

### Effects of disturbance on megafaunal assemblage composition

**Figure 5:**
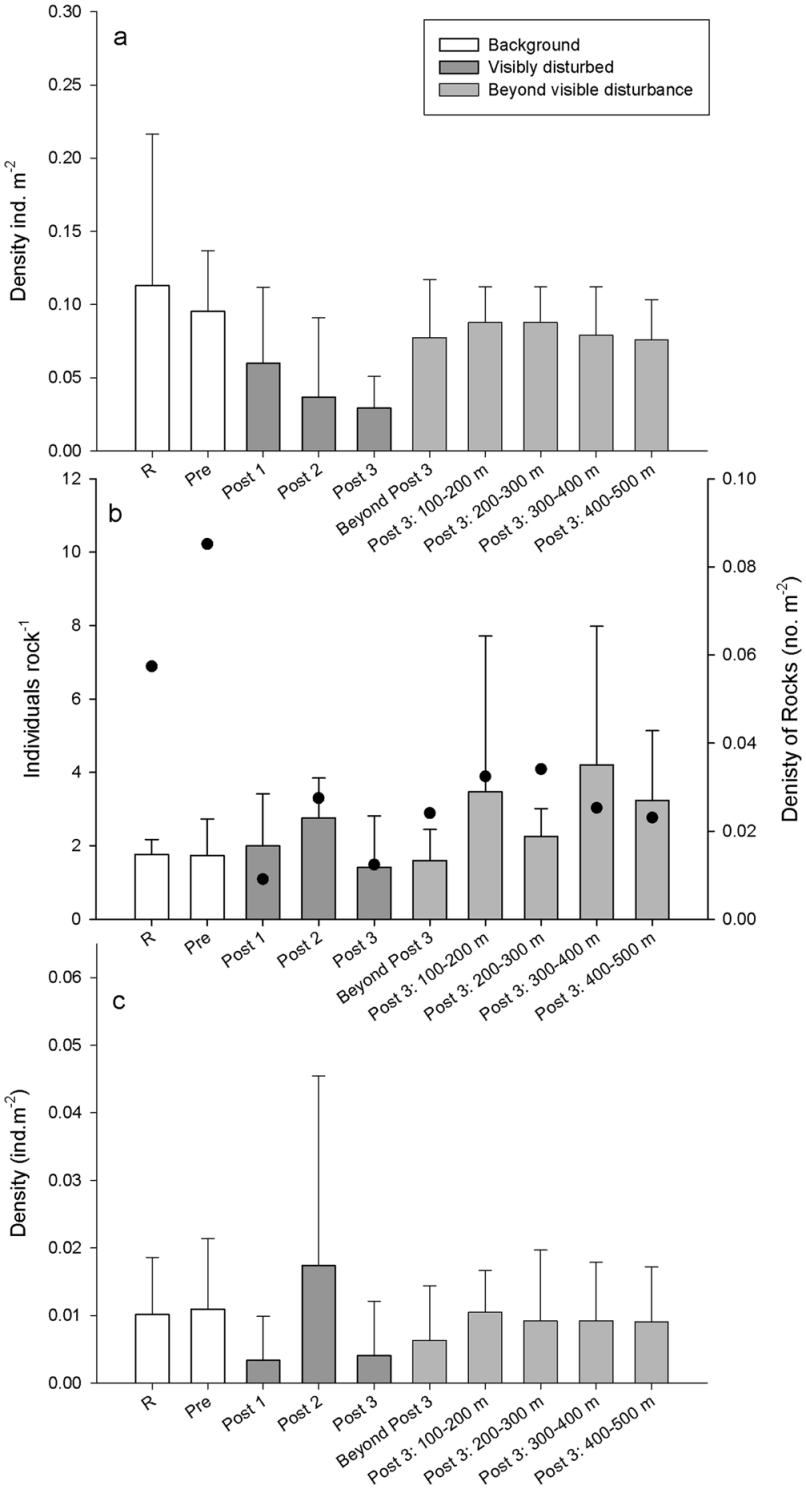
Mean (±sd) megafaunal invertebrate density (individuals m^−2^) at Morvin. (a) soft sediment, (b) hard substrate and (c) generalist megafauna. Background sites are shown in white, visibly disturbed areas in dark grey and areas beyond disturbance are shown in light grey. Filled circles in hard substratum chart present show the density of rocks in the transects.

There was variation in mean density of soft-substrate, hard-substrate and generalist megafauna between the sampling units (Figure 5). There was a significant main effect of distance (L-ratio_(d.f. = 5)_ = 27.703, p<0.001) and time (L-ratio_(3)_ = 25.362, p<0.001) on total density of benthic invertebrate megafauna at Morvin. There was, however, no significant interaction (L-ratio_(3)_ = 0.634, p = 0.889). Soft sediment invertebrate megafaunal density showed an effect of distance (L-ratio_(5)_ = 15.6, p<0.01) but no significant effect of time (L-ratio_(3)_ = 6.195, p = 0.1). There was no significant interaction (L-ratio_(3)_ = 0.785, p = 0.85). For the density of hard substrate invertebrates there was a significant main effect of time (L-ratio_(3)_ = 13.467, p = 0.004), but no significant effect of distance (L-ratio_(5)_ = 3.985, p = 0.552) or interaction between distance and time (L-ratio_(3)_ = 4.03, p = 0.258).

**Figure 6:**
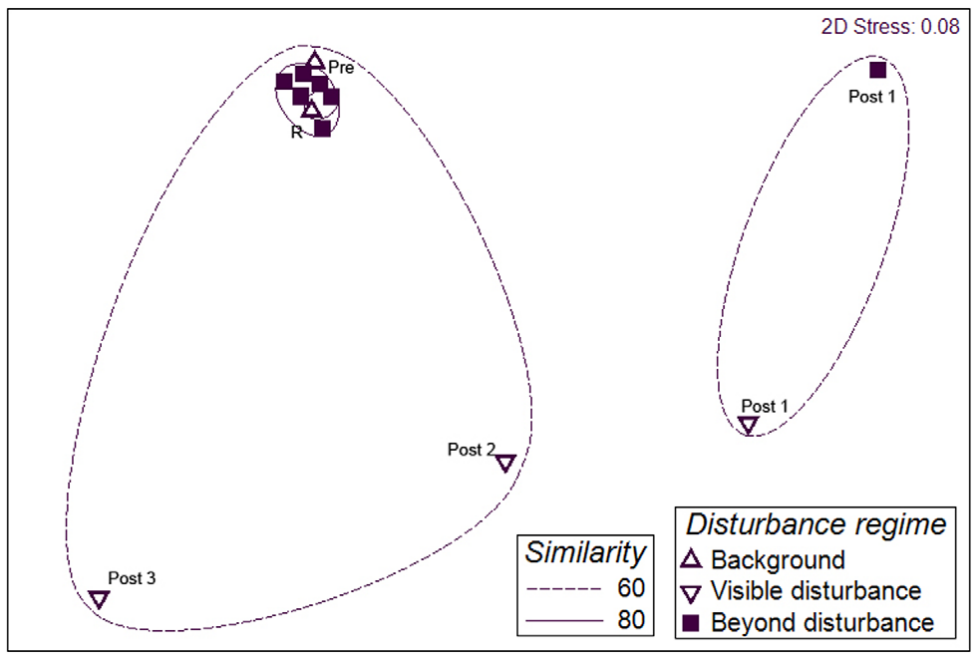
Multidimensional scaling ordination of megafaunal assemblages under different disturbance conditions. Based on Bray Curtis similarity of pooled invertebrate megafaunal density data for the disturbance zones in 2006 (Pre, Post 1 and Post 2) and 2009 (Post 3, R). For each survey the transects have been divided into Background, Visible Disturbance and Beyond Disturbance according to the coverage of the sediment by drill cuttings, notable groups are labelled. Similarity levels from cluster analysis.

PERMANOVA showed a significant effect of time (F_(3)_ = 0.163, p<0.001) but no significant effect of distance (F_(5)_ = 0.055, p = 0.163) or interaction (F_(3)_ = 0.021, p = 0.761). The multidimensional scaling plot (Figure 6) of the combined data for each transect disturbance/distance zone grouped the R and Pre sites and the sites beyond disturbance at the 80% similarity level.

It should be noted that the two-way design used here was limited by the lack of samples from distance zones greater than 100 m in all years except Post 3. There were only two distance zones for samples at most times, both within 100 m from the drilling activity. This limited replcation will reduce the ability to detect a main effect of distance or an interaction between distance and time in the statistical tests.

### Recovery

**Figure 7:**
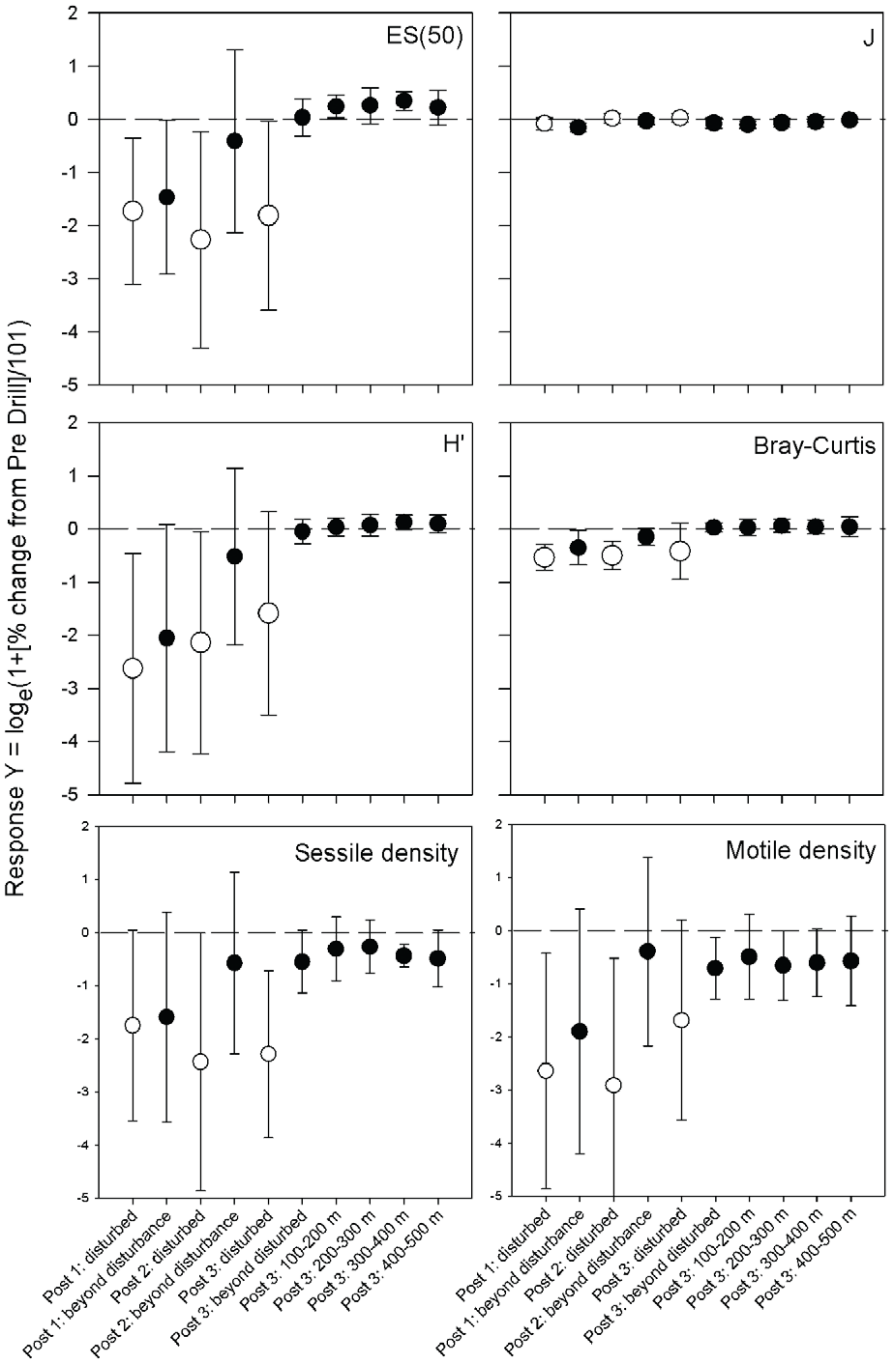
Response Y recovery index in comparison to pre-drill. Shown for Rarefied species richness (*ES_(50)_*), Species evenness (*J*), Shannon-Wiener Index (*H′*), Bray Curtis similarity, total sessile organism density and total motile organism density. Unfilled circles indicate disturbed zones and filled circles indicate distance from disturbance. Dashed lines indicate zero. Error bars = standard deviation.

For each of the indices tested, the transformed percentage difference from Pre-drill (Response Y) varied across the distance and time scales considered (Figure 7), but was generally more negative close to the disturbance event in both space and time. Response Y for the density of motile organisms showed no main effects of distance (ANOVA on ranks F_(5,70)_ = 2.135, p = 0.071), time (F_(2,70)_ = 1.253, p = 0.292) or the interaction (F_(2,70)_ = 1.297, p = 0.280). Response Y for the density of sessile organisms showed significant main effects of distance (F_(5,70)_ = 3.967, p<0.01), but no significant effect of time (F_(2,70)_ = 1.928, p = 0.153) or the interaction (F_(2,70)_ = 0.702, p = 0.499). Response Y for the Shannon-Wiener diversity and estimated richness (ES_50_) of megafauna revealled significant main effects of distance (H′: F_(5,70)_ = 14.116; ES_50_: F_(5,70)_ = 16.530; p<0.001 for both) and time (H′: F_(2,70)_ = 4.947, p<0.01; ES_50_: F_(2,70)_ = 4.027, p<0.05) and the interaction (H′: F_(2,70)_ = 3.349, p<0.05; ES_50_: F_(2,70)_ = 3.280, p<0.05). Response Y for the evenness of megafauna (J) had a significant main effect of distance (F_(5,70)_ = 4.275, p<0.05) but no significant main effect of time (F_(2,70)_ = 0.640, p = 0.530) or the interaction (F_(2,70)_ = 0.700, p = 0.502). Response Y for the Bray-Curtis similarity between megafaunal assemblages did not reveal any significant effects of distance (F_(5,70)_ = 2.068, p = 0.080), time (F_(2,70)_ = 2.551, p = 0.085) or the interaction (F_(2,70)_ = 0.310, p = 0.734).

### Evidence of biological activity

**Figure 8:**
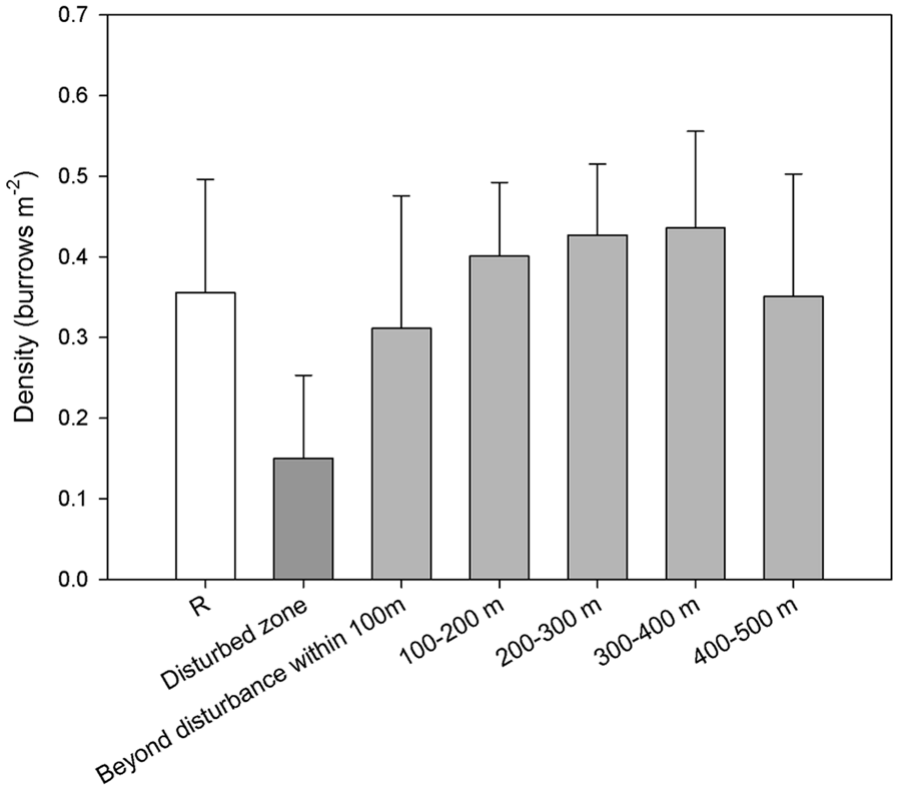
Mean density of decapod burrows (±sd) in the Post 3 survey at Morvin. White = Background, dark grey = Visible Disturbance, light grey = Beyond Disturbance.

Decapod burrows were common in the soft sediment at Morvin with mean densities of 3.5 m^−2^ in the Reference sites. Mean decapod burrow density differed significantly along the disturbance gradient in the 2009 surveys (Figure 8; ANOVA F_(6, 51)_ = 4.77, p<0.001). Pairwise comparisons (Holm-Sidak method) showed significant differences between the 2009 disturbed zone and all the other zones with the exception of the undisturbed area within 100 m of the well. The closest burrow was 5 m from the well and the numbers began to increase after 20 m distance.

## Discussion

### Background environment

At Morvin the rocks on the seabed provided heterogeneity in an otherwise soft-sediment environment. This is a typical situation for the northern North Atlantic [45]. The increase in habitat heterogeneity enhanced benthic diversity at Morvin, as has been shown elsewhere at global [46] and local scales [47]. The composition of the soft-sediment megafaunal assemblage was comparable to that found in areas of similar depth in the Porcupine Seabight (Table 3), southwest of Ireland, with species densities also similar [48], [49], [50], [51]. The available hard substratum at Morvin increased habitat heterogeneity with a resultant increase in density and species richness, most notably in the Porifera. In this respect there are direct similarities between Morvin and the megafauna from the Faroe-Shetland Channel to the south [52] and Le Danois Bank at equivalent depth in the Cantabrian Sea (Table 3) where exposed rock on an otherwise sandy seabed formed a distinct habitat with high abundances of the sponge *Phakellia ventilabrum* [53]. Of the demersal fish at Morvin, the presence of *Lophius piscatorius*, *Sebastes* sp. and *Chimera monstrosa* were consistent with results from previous studies of Norwegian shelf-edge Atlantic water [54].

**Table 3:** Mean taxon densities (numbers 100 m^−2^) of species shared between Morvin and other Atlantic sites at similar depth.

Taxon	Morvin	Le Danois Bank	Porcupine Seabight	West of Shetland
*Hymedesmia* sp.	0.42	5.67		0.73
*Phakellia sp.*	0.08	1.02		
*Kophobelemnon stelliferum*	5.63		260	
*Funiculina* sp.	1.74	0.04 (T)		
*Cerianthus* sp.	0.39	0.638		
*Colus sp.*	0.14	0.22 (T)	X	
*Geryon* sp.	0.28	0.03(T)	X	0.72
*Pandalus* sp.	0.42		X	1.65
*Munida* sp.	0.14	0.829	X	7.91
*Parastichopus tremulus*	2.62	0.191	X	7.69
*Henricia* sp.	0.2		X	1.61
*Ceramaster* sp.	0.26			1.32
*Echinus* sp.	0.14	0.04 (T)		2.93
*Asterias rubens*	X		X	0.29
*Porania* sp.	0.2	0.32		0.55
*Cidaris cidaris*	0.14	0.01		11.43
No of taxa in common with Morvin		11	8	11

Morvin (this study) the highest of pre and R densities are presented. Le Danois Bank, northern Spain (425–550 m depth); data from photographic study by [53] augmented, if additional species were encountered, with trawl data (marked with (T); [70]). Porcupine Seabight, SW Ireland (150–550 m depth) data from [49] and unpublished data (Brian Bett, NOC). West of Shetland, UK (420 to 508 m depth); data from [52]. If species were found, but not enumerated, presence was marked with X. The final row represents the total number of taxa common to Morvin and the relevant literature study.

It is important to evaluate disturbance-related changes at Morvin within the context of the broader temporally-dynamic ecosystem. Temporal change in the deep sea is receiving increasing attention [55] and recent studies have shown seasonal and interannual changes in benthic megafaunal communities in the deep north-east Atlantic [29], [56], [57], [58]. Shallow-water studies of recovery trajectories have identified seasonal recruitment as an important factor [59]. At Morvin the megafauna in the Pre-drill (23^rd^ March 2006) and R site surveys (3–4^th^ May 2009) showed no significant differences in density, diversity or assemblage composition. This suggests that there was limited natural temporal change at the time-scale analysed, thus supporting comparisons between pre- and post-drilling surveys.

### Initial disturbance

Recent studies in the north-east Atlantic have revealed drill cuttings extending to approximately 200 m from the well with reduced megafaunal density and diversity within the disturbed area [4], [15]. In the present case, the visible extent of the cuttings reached beyond 100 m from the BOP to the north-west but were generally less than 100 m. This equates to an area of at least 26601 m^2^. This is considerably smaller than reported in older studies of exploration wells in the north-east Atlantic in which oil based drilling mud was used and there was less regulation for discharges to the seabed [20], [60]. The extent was also smaller than reported in more recent studies at a similar depth in the Faroe-Shetland Channel (>66800 m^2^) [4], albeit at a site with multiple wells drilled. The persistance of the effects of water based mud and drill cuttings on megafauna is unknown and the increasing number of wells in a field could result in larger areas being affected, with potential accumulating or synergistic long-term effects.

The drill cuttings deposited at Morvin caused an initial physical disturbance, which resulted in smothering of the benthic fauna. The longer-term impacts associated with such an event include the possible chemical effects of the drilling mud, hypoxia related to chemicals in the drilling mud or to smothering, and reduced habitat heterogeneity caused by the rapid creation of a smooth, soft-sediment environment. In terms of the physical nature of the disturbance caused by exploration drilling, there are similarities with the disposal of dredged material [61] and bottom trawling [62], [63].

### Persistence of the disturbance

Although there was still visible evidence of disturbance surrounding the well in 2009, the total area visibly disturbed by cuttings deposition had decreased considerably since 2006. Then, the cuttings pile was over 400 mm deep at 10 m distance from the well and at 50 m there was a thin covering of unevenly distributed drill cuttings, estimated to be less than 50 mm. Although the area of deeper cuttings coverage was the most impacted area in this study, the area with the thinner layer of cuttings can not be discounted as even a thin layer of cuttings may affect the sediment bacteria and smaller size fractions of benthic fauna. These organisms were not visible in the video methods used in this study but have important roles in the functioning of benthic ecosystems as well as providing food source to some megafaunal organisms. An elevated “crater” remained at the exact well location which attracted increased abundance of the fish *Sebastes* sp. (excluded from the quantitative analysis). The increased quantity of cuttings deposited close to the well, and the cement used to secure the structure of the well in the plug and abandon phase [64], may consolidate the cuttings pile in the immediate vicinity of the well. It has been suggested that, unless disturbed, cuttings piles remain relatively unchanged over time [10] and that the cuttings further from the well may be stable [65]. As a result, the obscurring of the disturbed sediment by the natural settlement of material from the water column may be a more important factor in reducing the visible extent of the cuttings than the erosion and lateral transport of the deposited drill cuttings by the currents. Indeed, large accumulations of sediment on coral reefs in the Morvin area [34] suggest relatively high sedimentation rates. However, lateral transport and the resulting breakdown of cuttings piles has been suggested by the presence of barite particles incorporated into the skeletons of corals located 4 km away from a 20 year old exploration well elsewhere in the Norwegian Sea [66].

Barium levels at Morvin were elevated, indicating persistance of the drill cuttings after three years. Although Ba is considered non-toxic, there remains debate in relation to the use of barite as a weighting agent in drilling mud. A variety of sublethal effects have been reported from laboratory studies such as reduced condition (gill damage) in benthic bivalves [67] and lower colonization by macrofauna of sediment treated with barite [68]. Other studies suggest the deposition of barite results in changed physical properties of the sediment [8], which in turn may alter habitat heterogeneity and increase meiofaunal density, as shown in a laboratory study [69]. The most abundant motile organism at Morvin, the holothurian *Parastichopus tremulus*, was completely absent from the disturbed areas of the post-drilling surveys in 2006. Seasonal variations in the density of *P. tremulus* are known [70] but owing to the relatively short time period between the Pre-drill survey and the first Post-drill survey, and consistent abundance of *P. tremulus* at the same time of year in 2009 outside the disturbed zone, it is likely that this species was absent because of the disturbance. This could be either because holothurian distribution is determined by food particle availability [51], which may be reduced on the newly deposited cuttings, or because holothurians ingest food particles selectively [71] and may therefore avoid consuming the cuttings which consist of differing physical properties [8] to the background sediment.

### Megafaunal recovery

There does not appear to be differential recovery between the visible disturbance zones within 100 m of the well (an interaction between distance and time factors), although these tests were limited by low replication. However, at a finer scale abundance was still reduced in the immediate vicinity of the well in the Post 3 survey.

Within 100 m of the drilling there were detectable differences in total megafauna between the visibly disturbed and not visibly disturbed areas. Most of this variation appeared to be within the sessile fauna. In comparison to the sites further from disturbance in 2009 there was increased variability in the samples close to the source of disturbance both spatially and temporally. Increased variability has been discussed as an indication of stress in marine communities [72]. In terms of the benthic megafauna, the most notable difference in the community structure between the 2009 disturbed zone and the reference sites was the reduction in sessile organisms. After the drilling operations, the dominant sponges on the hard substrata (*Phakellia* sp. and *Mycale* sp.) were rare, primarily because of burial of their habitat. Further research is required to determine how sponges respond to lower degrees of sedimentation leading to partial burial. Throughout the study pennatulids were the most common organisms on the soft sediment. Their numbers were low in the visibly disturbed area in 2009. Pennatulids are slow growing and may therefore take considerable time to recover from disturbance [73]. The larval recruitment and settlement rates for these organisms are unknown. Studies on the reproduction of *Kophobelemnon stelliferum*, *Pennatula phosphorea* and *Funiculina quadrangularis* suggest these species have lecithotrophic larvae, which may remain in the water column until suitable habitat is located [49], [74], [75] and could possibly avoid settlement on sediment disturbed by drilling mud and cuttings.

Bioturbation rates are poorly understood in deep water but are important indicators of ecosystem function. This process is evidently important in the recovery of soft sediments after physical disturbance. In the Post 3 survey, large burrows were present on the disturbed seabed, indicating activity of the decapod *Geryon* sp. in this area. These crabs were observed entering and leaving these burrows, the structure of which was very similar to *Geryon trispinosus* burrows on the seafloor of the Porcupine Seabight [48]. This activity is likely to be important in the re-distribution of the sediment and gradual breakdown of the cuttings pile. The nearest burrow was 5 m from the well indicating activity in this area in the three years since disturbance. The presence of new burrows and the apparent longevity of some *Lebenspuren* [76] implies that reduced burrow density may not necessarily indicate long-term reduction in bioturbation activity. The holothurian *Parastichopus tremulus* is important in horizontal dispersal of sediment [51] and therefore, potentially, in the re-distribution of cuttings and drilling mud. However, the Morvin data suggest *P. tremulus* avoids feeding on the cuttings and thus probably does not contribute much to the re-distribution of sediments. Although not considered in this study, the inclusion of the macrofauna, which may be more abundant than the megafauna both numerically and in terms of biomass and which include important bioturbators, would benefit future studies of recovery. Indeed, experimental data suggest that macrofaunal assembalges may colonize water based drilling mud rapidly [77]. In addition, the chemical disturbance and altered sediment characteristics may also affect meiofaunal assemblage composition [78], [79] and the microbial assemblage, which could influence food availability and therefore the recovery of the larger benthic fauna.

Studies on the Georges Bank, Gulf of Maine (60–100 m depth) suggest limited effects of oil and gas exploration activities on megafauna (at finer-scale resolution than Morvin) and evidence of recovery by the macrofauna [80]. The Georges Bank is subject to high energy storms that redistribute sediments. In contrast, at a lower energy abyssal site experimental disturbances designed to predict the effects of nodule mining [23] showed limited evidence for recovery of the megafauna after seven years with no subsequent disturbance. It has been suggested that recovery is complicated and influenced by factors including the scale of the disturbance [81], the type and frequency of disturbance and the local environmental conditions [62], [82]. These factors complicate the assessment of recovery in studies such as this one, limited by operational contraints (access to a deep site, spatial reach of the ROV in the earlier surveys) and highlight the importance of suitable spatial and temporal replication. To address this issue, bioequivalence methods have been used to assess ecological impacts [83] but have not been universally adopted in ecological studies [84].

The limited and ambiguous data on benthic recovery in deeper water highlight the need for more studies. At present, differences in the physical and biological environments at different study sites and the individual nature of each cuttings pile make it impossible to draw general conclusions. A similar study of a drilling site in the Faroe-Shetland Channel [85] has also revealed a small area of reduced faunal density and diversity close to the well after three years. We suggest that the significant decrease in megafaunal density, which appears to persist for at least 3 years at both sites will occur at all deep-water drilling sites, with the severity of the impact likely to be correlated with the amount of material deposited on the seabed and the local environmental conditions. It is anticipated that the effect will be greater in deeper, colder areas, where the rate of metabolism and growth are expected to be considerably lower [76], thereby reducing the rate of recovery. The change in sediment particle size may also retard recovery, as demonstrated in shallower water [86]. With increasing anthropogenic activity in deeper waters it is essential to understand the initial effects on benthic fauna and their recovery to such impacts. Hydrocarbon exploration disturbance provides a valuable tool to study disturbance and recovery trajectories in remote deep-water habitats, which are generally difficult to access.

## Supporting Information

Table S1 **Video grabs from the video transects to the south of the well in the first post-drill survey in 2006 and the recovery survey in 2009.**
(DOCX)

Figure S1 **Burrows in the soft sediment at Morvin.** Decapod crustaceans, likely *Geryon* sp. were often seen entering these burrows.
(DOCX)
