# Supplementary material for: Recovery of Benthic Megafauna from Anthropogenic Disturbance at a Hydrocarbon Drilling Well (380 m Depth in the Norwegian Sea)
Source: PLoS One. 2012 Oct 8;7(10):e44114. doi: 10.1371/journal.pone.0044114 (PMC3466215; doi:10.1371/journal.pone.0044114)
Supplement: Table S1 — Video grabs from the video transects to the south of the well in the first post-drill survey in 2006 and the recovery survey in 2009. (DOCX) [file pone.0044114.s001.docx]

Table S1: Video grabs from the video transects to the south of the well in the first post-drill survey in 2006 and the recovery survey in 2009.

| Position | Distance to well (m) | Post-drill 1 (2006) | Post-drill 3 (2009) |
| --- | --- | --- | --- |
| 380172  7224481 | Well location | 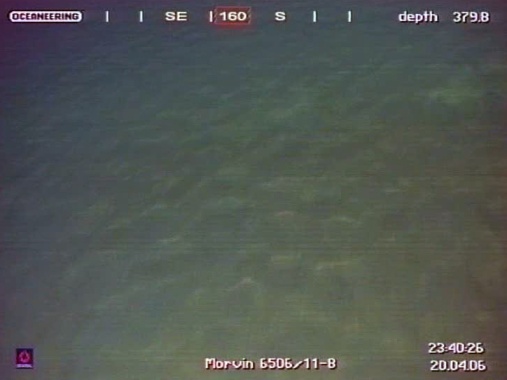 | 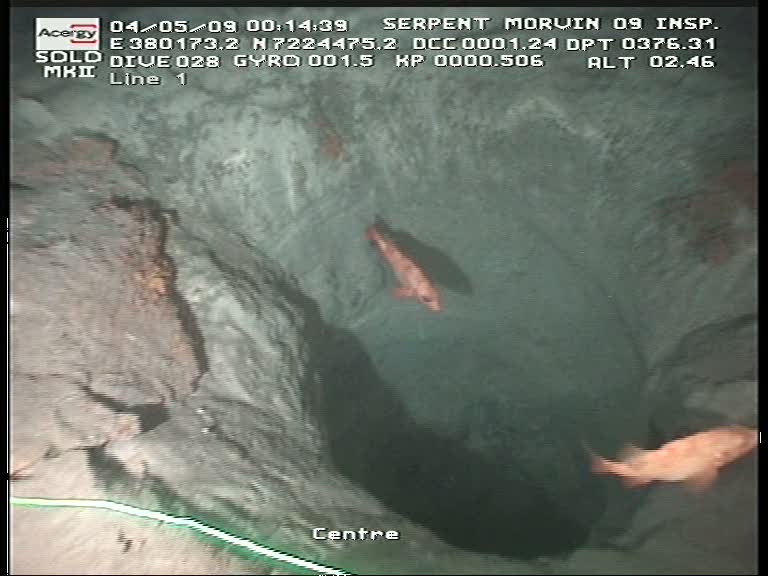 |
| 380172  7224471 | 10 | 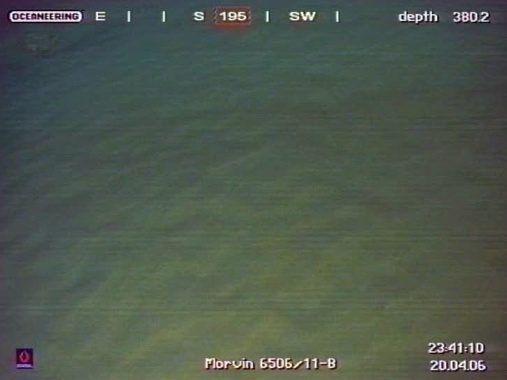 | 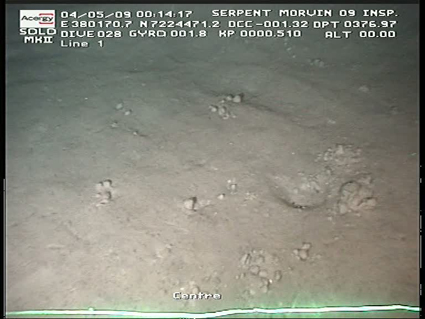 |
| 380172  7224463 | 18 | 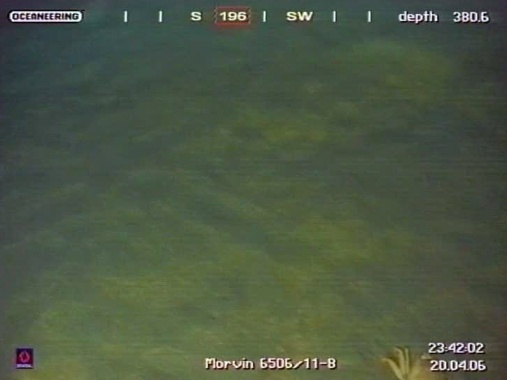 | 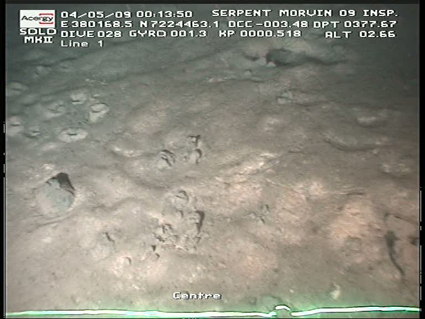 |
| 380172  7224441 | 40 | 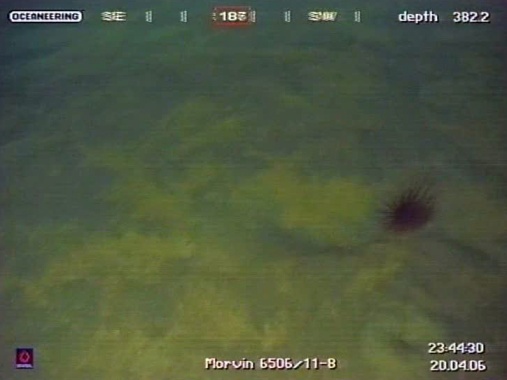 | 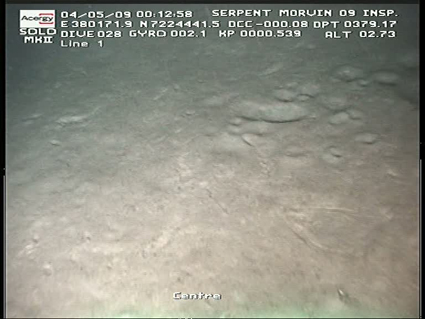 |
| 380172  7224418 | 63 | 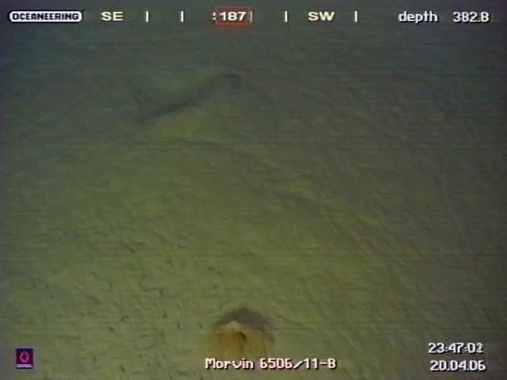 | 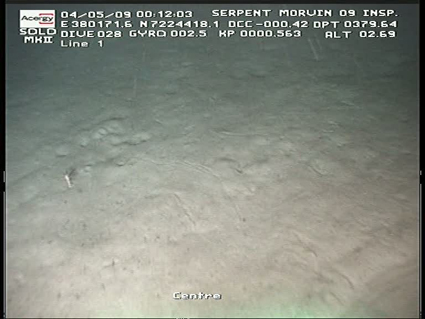 |
| 380172  7224400 | 81 | 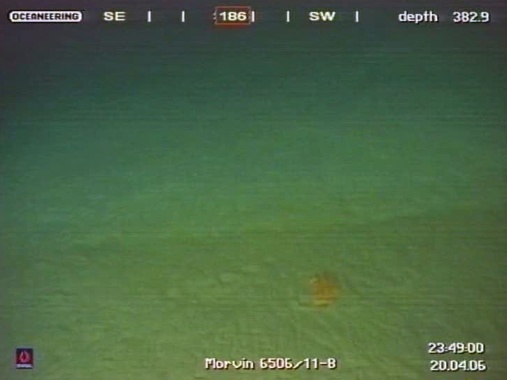 | 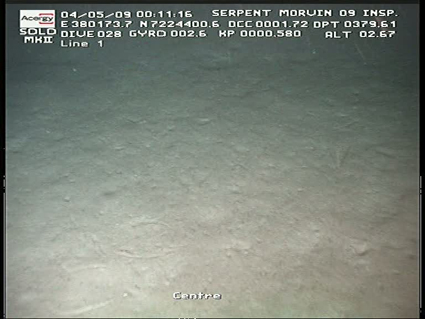 |
| 380172  7224381 | 100 | 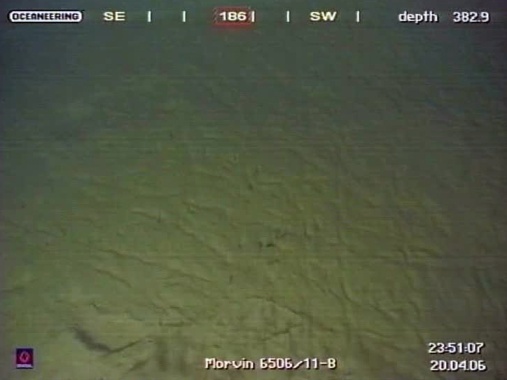 | 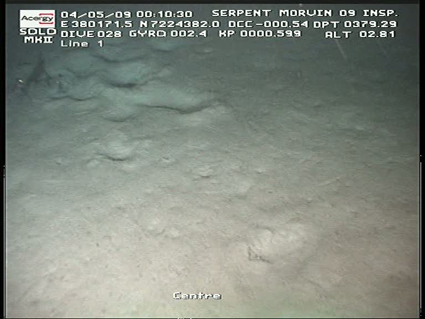 |
