# Supplementary material for: Recovery of Benthic Megafauna from Anthropogenic Disturbance at a Hydrocarbon Drilling Well (380 m Depth in the Norwegian Sea)
Source: PLoS One. 2012 Oct 8;7(10):e44114. doi: 10.1371/journal.pone.0044114 (PMC3466215; doi:10.1371/journal.pone.0044114)
Supplement: Figure S1 — Burrows in the soft sediment at Morvin. Decapod crustaceans, likely Geryon sp. were often seen entering these burrows. (DOCX) [file pone.0044114.s002.docx]

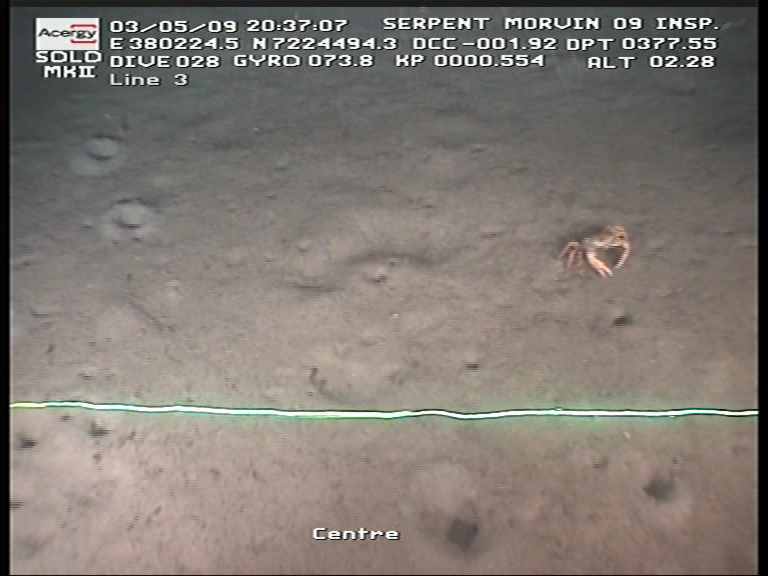

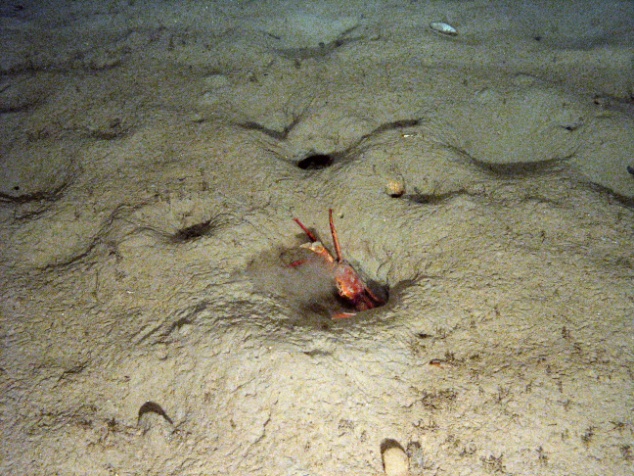


Figure S1: Burrows in the soft sediment at Morvin. Decapod crustaceans, likely *Geryon* sp. were often seen entering these burrows.
